# Olfactory dysfunction is common in classical infratentorial superficial siderosis of the central nervous system

**DOI:** 10.1007/s00415-022-11329-y

**Published:** 2022-08-23

**Authors:** Natallia Kharytaniuk, E. A. Lim, E. Chan, M. Pavlou, D. J. Werring, D. E. Bamiou

**Affiliations:** 1 Ear Institute, University College London, London, UK; 2 National Institute for Health and Care Research, University College London Hospitals Biomedical Research Centre (Deafness and Hearing Problems Theme), London, UK; 3 Department of Neuro-Otology, Royal ENT and Eastman Dental Hospitals, London, UK; 4 Lysholm Department of Neuroradiology, National Hospital for Neurology and Neurosurgery, Queen Square, London, UK; 5 Department of Imaging, Imperial College Healthcare NHS Trust, London, UK; 6 Department of Neuropsychology, National Hospital for Neurology and Neurosurgery, Queen Square, London, UK; 7 Stroke Research Centre, Department of Brain Repair and Rehabilitation, Queen Square Institute of Neurology, University College London, London, UK; 8 Department of Statistical Science, University College London, London, UK; 9 Department of Neurology, National Hospital for Neurology and Neurosurgery, Queen Square, London, UK; 10 Centre for Auditory Research, UCL Ear Institute, 332-336 Gray’s Inn Road, London, WC1X 8EE UK

**Keywords:** Superficial siderosis, Central nervous system, Smell test, Olfaction, Hearing, Cognition

## Abstract

**Background:**

Established features of classical infratentorial superficial siderosis (iSS) include hearing loss, impaired balance, myelopathy and, less commonly, cognitive compromise. Olfactory function may be affected but dedicated studies are lacking. This study aimed to assess the prevalence of olfactory dysfunction in iSS and correlate it with auditory and cognitive functions.

**Methods:**

Ten participants with iSS completed the University of Pennsylvania Smell Identification Test (UPSIT). The scores were compared with population norms; regression analysis was performed to evaluate associations between the scores and hearing thresholds (3-frequency average, 3FA) or the number of cognitive domains impaired. Imaging was reviewed for haemosiderin distribution and to exclude other causes of olfactory and hearing dysfunction.

**Results:**

Eight of ten participants were male; the mean (standard deviation, SD) age was 52.5 (14.5) years. Olfactory hypofunction was identified in all participants and in six (60%) was moderate or completely absent. The mean UPSIT score of 25.5 (7.8) was significantly worse than population norms (difference in means − 10.0; 95% CI − 15.6 to − 4.4). Linear regression identified an association between UPSIT and hearing thresholds (*R* = 0.75; *p* = 0.013). The score decreases by 0.157 units (95% CI − 0.31 to − 0.002; *p* = 0.048) per unit increase in 3FA, after adjusting for hearing loss risk factors. There was no statistically significant association between UPSIT and cognitive function (*R* = 0.383; *p* = 0.397).

**Conclusion:**

We report a high prevalence of olfactory dysfunction in iSS, the severity of which correlated with hearing loss. Olfaction appears to be a core feature of the iSS clinical syndrome that should be assessed routinely.

## Introduction

Established core features of infratentorial (classical) superficial siderosis (iSS) are hearing impairment and imbalance whilst other features including myelopathy and cognitive dysfunction are also recognised [1–4]. iSS is characterised by chronic low volume extravasation of blood into cerebrospinal fluid (CSF) often from a dural defect following head or spinal trauma or surgery [2, 5]. Iron degradation is facilitated by microglia and Bergmann glia resulting in haemosiderin deposition in the subpial layers of the central nervous system (CNS), best visualised on paramagnetic resonance imaging sequences [5, 6]. Saturation of the iron degradation pathway leads to lipid peroxidation from free iron species and ultimately neuronal damage and gliosis [2, 6, 7]. Halting the effects of the neurotoxic iron—and thus associated functional decline including worsening olfaction—underpins the current principles of iSS treatment which includes surgical repair of dural defect where identified, or with an iron chelating agent (e.g. deferiprone) although the evidence for efficacy of these approaches is limited [8–10].

There is invariable involvement of infratentorial structures and particularly the cerebellum, likely due to the abundance of Bergmann glial cells that synthesise ferritin, and constant exposure of the cerebellum to renewed haemorrhagic CSF due to its early irrigation [6, 11]. The vestibulocochlear nerve (CNVIII) can be affected due to its exposure to CSF along its glial portion in the pontine cistern [2, 5, 7]. The olfactory tract and bulb may also be exposed to haemorrhagic CSF near the cribriform plate [2, 7, 11, 12] and their involvement in siderosis has also been described [2, 6, 7, 11] yet olfactory loss in iSS has not been systematically studied [2–4]. The aims of this study were thus to: (1) assess olfactory function in individuals with iSS; and (2) determine if association exists between olfactory impairment and hearing and cognitive functions which are known to be frequently affected in iSS.

## Methods

### Ethics permissions, study consents and data management

The study was performed in line with the principles of the Declaration of Helsinki. It was approved by the National Health Service Health Research Authority and Health and Care Research Wales Research Ethics Committee (REC 19/LO/1162 AM01). All study participants provided formal written consent for the study. To anonymise the study participants’ details due to the small study sample, we report aggregated results.

### Study setting and participants’ recruitment

We invited seventeen patients with radiologically confirmed iSS using the diagnostic radiological criteria [5] and who have been under the care of the Queen Square National Hospital for Neurology and Neurosurgery superficial siderosis multidisciplinary team to participate in the study. Thirteen smell identification kits were distributed by post between November 2020 and June 2021.

### Data collection

#### Demographics

We recorded participants’ demographics, smoking history and presence of additional risk factors for olfactory loss and hearing loss. Likely causative events for development of iSS were known in all cases except one and included history of surgery to spine or posterior fossa, trauma to spine with blunt force and spontaneous intracranial hypotension; these were all reviewed and excluded as a likely direct cause for olfactory dysfunction because the injuries were not expected to injure olfactory pathway structures, particularly the olfactory bulb or nerve. History of mild head trauma was recorded in three cases but was not deemed the cause for iSS. There was no history of iatrogenic injuries including anterior cranial base craniotomies or neoplasms involving olfactory mucosa or frontal lobe. We calculated disease duration from the time of the likely causative event where known or from the time of diagnosis (one case).

#### Olfactory function

Olfactory function was assessed using the University of Pennsylvania Smell Identification Test (UPSIT, British version), which is a forced-choice 40-item self-administered test that contains four booklets with strips of embedded microencapsulated odours released by scratching the strips. The scores were calculated using manufacturer’s answer key as the sum of correct answers, and compared to the age- and gender-matched norms [13].

#### Other clinical parameters

Hearing, cognitive and neuroimaging evaluation was performed as part of the patients’ clinical care.

Hearing function was assessed using pure-tone audiometry following standard operating procedure in line with the British Society of Audiology guidelines [14]. The hearing levels were represented by 3-frequency averages (3FA: 0.5/1/2 kilohertz, kHz) for both ears; 120 decibel hearing level (dBHL) values were substituted to calculate 3FA at frequencies where the hearing thresholds were not reached. Higher 3FA values represented worse hearing levels. Pure-tone audiometry was performed within (median, interquartile range IQR) 4 (5) months of UPSIT.

Magnetic resonance imaging (MRI) of the brain, including paramagnetic sequences, was either performed at one of the Trust’s imageing sites or at the participants’ local facility (three cases) and in which cases the images were migrated using secure online clinical imageing exchange portal (Sectra IEP, UK).

Axial T2-weighted MR images were reviewed to exclude any secondary causes for reduced olfaction, such as chronic rhinosinusitis with or without nasal polyposis which may contribute to olfactory hypofunction, and included the following regions: nasal and para-nasal cavities, olfactory bulb and tract, cribriform plate, orbito-frontal regions [15, 16]. Additionally, regions representing hearing function, such as cerebellopontine angles (CPA), internal acoustic meati (IAM) and cochleae bilaterally, were reviewed to exclude any secondary causes for hearing dysfunction.

Paramagnetic sequences, which included susceptibility-weighted (SW) or T2* gradient-recalled echo (T2*GRE) images, were reviewed for the presence of haemosiderin, and included orbito-frontal regions, frontal and temporal lobe convexities, Sylvian fissures and vestibulocochlear nerve complexes. All imaging had been previously reviewed by the clinical neuroradiology team and was co-reviewed by the senior neuroradiology fellow (EAL) in the context of this study. Imaging was performed within (mean, standard deviation SD) 8 (6) months of UPSIT.

Cognitive function was assessed by the clinical neuropsychology team, within (mean, SD) 21 (10) months of UPSIT; cognitive function of one participant had been reported in our previous study [1]. The test battery had been described elsewhere [1] and compared against respective standardised normative data [1]. Cognitive impairment in a domain was defined as scoring at or below the fifth percentile on any one test in that domain.

### Statistical analysis

Statistical analysis was performed using SPSS (v27, IBM Corporation, Armonk, NY). Frequencies and percentages were reported for categorical data. Continuous data were assessed for normality using Shapiro–Wilk test. Significance levels were set at 0.05. For normally distributed data, mean and SD were reported; difference in means was assessed using Student’s *t*-test. Median and IQR were reported for continuous data that appeared to deviate from normality. Unadjusted linear regression was performed to determine the association between UPSIT scores and hearing loss or cognitive dysfunction, with unit change between two variables in each model represented by *b*-coefficient.

## Results

Results from ten completed test kits were included in this study. The participants’ median age was 58.5 (27.8) years; 80% were males. The mean disease duration was 21.4 (12.6) years. History of rhinitis or smoking was present in 60% of participants. No participants reported any symptoms or prior history of COVID-19.

Risk factors for hearing loss (in addition to iSS) were present in 50% of participants, and in 40% prior to the likely causative event.

UPSIT scores indicated olfactory hypofunction in all participants, 30% of whom had moderate hypofunction and another 30% of participants had anosmia (Table 1), with 40% participants symptomatic for olfactory dysfunction.

**Table 1:** Participants’ outcomes

Case	UPSIT	Orbito-frontal region		Frontal lobe convexity		Sylvian fissure		CNVIII	
	Category								
		Rt	Lt	Rt	Lt	Rt	Lt	Rt	Lt
1	Moderate or worse	** + **	** + **	** + **	** + **	** + **	** + **	** + **	** + **
2	Moderate or worse	** + **	** + **	** + **	** + **	** + **	** + **	** + **	** + **
3	Moderate or worse	** + **	** + **	** + **	** + **	** + **	** + **	** + **	** + **
4	Moderate or worse	** + **	** + **	** + **	** + **	** + **	** + **	** + **	** + **
5	Mild	** + **	** + **	** + **	** + **	** + **	** + **	** + **	** + **
6	Mild	** + **	** + **	** + **	** + **	** + **	** + **	** + **	** + **
7	Mild	–	** + **	–	** + **	–	** + **	** + **	** + **
8	Moderate or worse	–	–	** + **	** + **	–	–	** + **	** + **
9	Moderate or worse	–	–	–	–	** + **	** + **	–	–
10	Mild	–	–	–	–	–	–	** + **	** + **

University of Pennsylvania Smell Identification Test (UPSIT) results, represented by categories (derived from the manual [13]): anosmia, moderate or mild microsmia were identified; and magnetic resonance imaging (MRI) findings, which included paramagnetic sequences (represented by susceptibility-weighted imaging, SWI or T2* gradient-recalled echo, T2*GRE), of olfactory and auditory regions; CNVIII 8th cranial nerve

*Lt* left, *Rt* right, “ + ” haemosiderin observed, “–” haemosiderin not observed

**Fig. 1:**
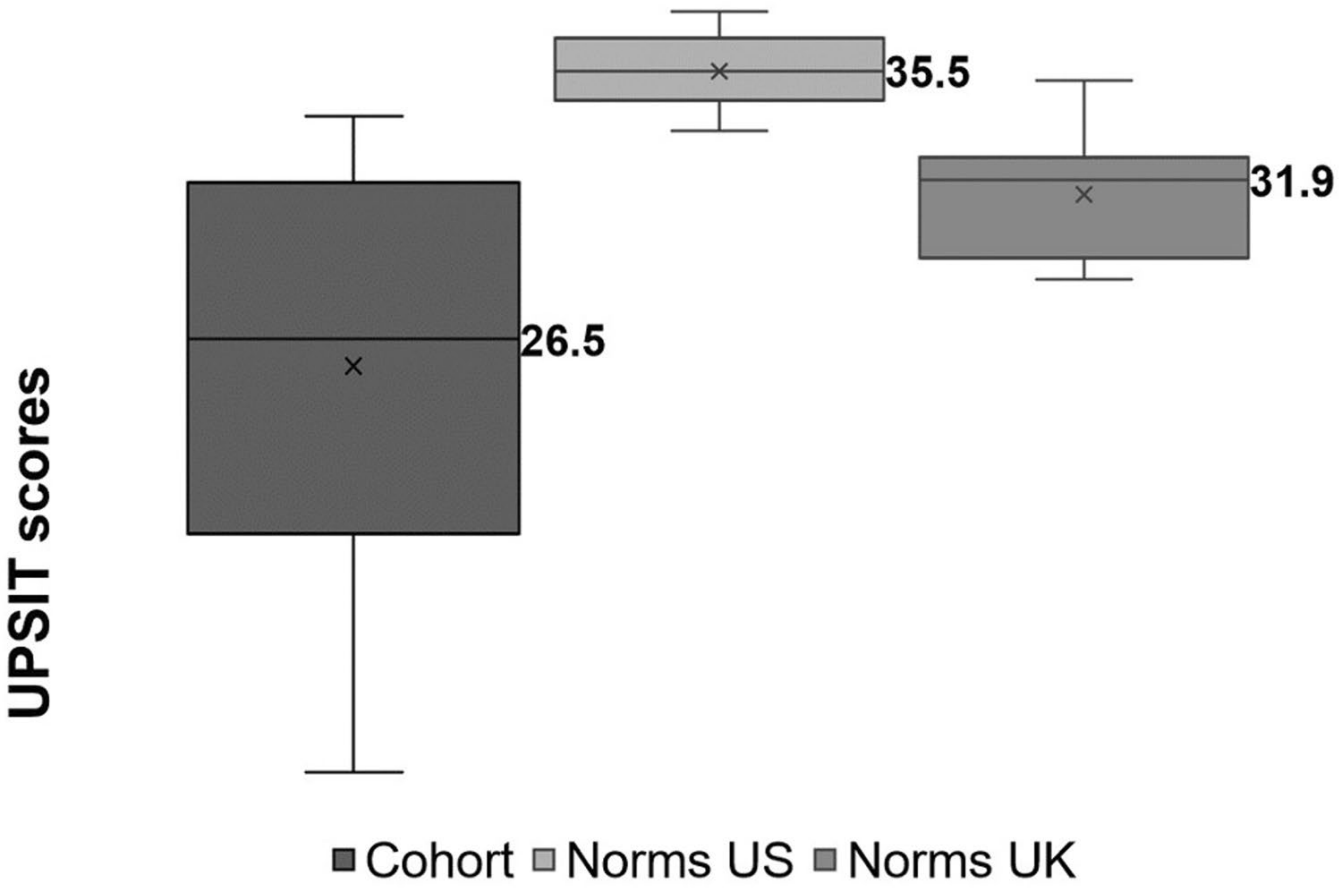
University of Pennsylvania Smell Identification Test (UPSIT) scores for the study cohort, calculated as the sum of correct answers (to the maximum best of 40) and compared with age- and gender-matched norms [13] and with a healthy English population sample; *x* = mean

The mean score was 25.5 (7.8) (Fig. 1) and did not statistically significantly differ between females and males (difference in means 8.8; 95% CI − 4.5 to 22.0; *p* = 0.165).

The scores were significantly worse than age- and gender-matched norms provided within the test manual (difference in means − 10.0; 95% CI − 15.6 to − 4.4; *p* = 0.003). The scores were also worse than age- and gender-matched norms derived from a healthy English population sample of 212 individuals in Ipswich (difference in means − 5.8; 95% CI − 11.4 to − 0.1; *p* = 0.047).

Hearing thresholds were significantly worse (higher) for participants with risk factors for hearing loss than without the risk factors (difference in means 50.3; 95% CI 4.3 to 96.4; *p* = 0.036).

Linear regression identified a strong association between UPSIT scores and the hearing thresholds (*R* = 0.746; *b* = − 0.145; 95% CI − 0.3 to − 0.04; *p* = 0.013); hearing thresholds accounted for 55.7% (adjusted *R*^2^ = 50.2%) of variability of UPSIT scores in the regression model (Table 2). After adjusting for the presence of risk factors for hearing loss, a strong association between UPSIT scores and hearing thresholds remained (*R* = 0.749; *b* = − 0.157; 95% CI − 0.31 to − 0.002; *p* = 0.048), with hearing thresholds accounting for 56.1% (adjusted *R*^2^ = 43.6%) of UPSIT scores variability.

**Table 2:** Unadjusted linear regression analyses between UPSIT scores and participants’ age, hearing thresholds (represented by 3-frequency average, 3FA, for both ears and uncorrected and corrected for risk factors for hearing loss, RF HL), and cognitive function (represented by the number of  impaired cognitive domains)

Unadjusted regression analysis for UPSIT scores	*b*	95% CI for *b*
Age (*n* = 10)	− 0.103	− 0.53 to 0.32
Hearing loss (3FA) (*n* = 10)	− 0.145	− 0.25 to − 0.04
Hearing loss (3FA) corrected for RF HL (*n* = 5)	− 0.157	− 0.31 to − 0.002
Number of cognitive domains (*n* = 7)	− 1.623	− 6.13 to 2.88

The level of significance was set at 0.05 (2-tailed); 95% confidence intervals (CI) for *b*-coefficient included

Cognitive assessment results were available for 7/10 (70%) participants. All participants had some degree of impairment; visual memory and executive functions were both the most frequently affected domains (in 5/7, 71%). Two out of seven (29%) patients reported severe symptoms of anxiety and depression on a formal scale.

There was no statistically significant association between UPSIT scores and the number of impaired cognitive domains (*R* = 0.383; adjusted *R*^2^ = − 0.024; *b* = − 1.62; 95% CI − 6.1 to 2.9; *p* = 0.397) (Table 2).

**Fig. 2:**
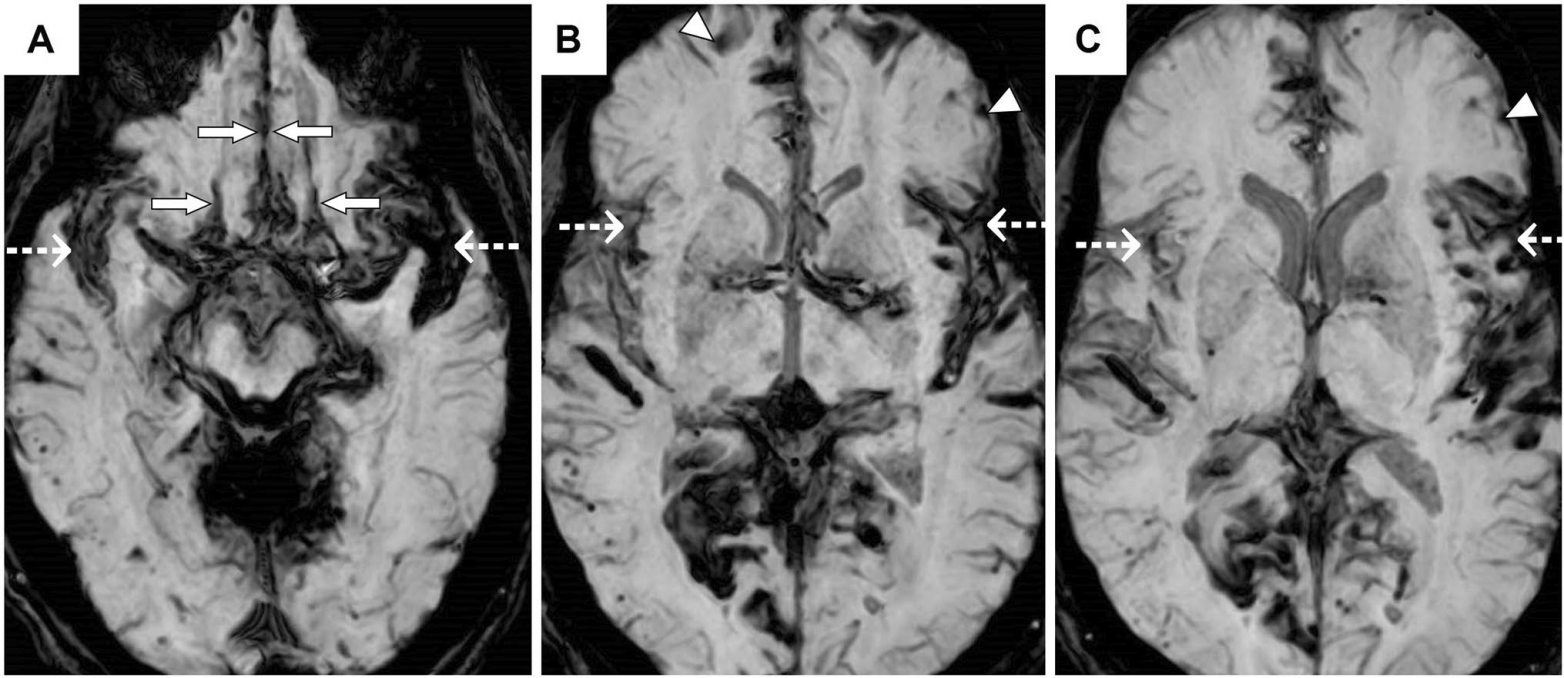
Axial susceptibility-weighted magnetic resonance images of the brain demonstrating haemosiderin deposits lining the orbito-frontal regions (marked with solid arrows, **A**), Sylvian fissures (marked with dashed arrows, **A**–**C**) and the frontal lobe convexities (marked with arrowheads, **B**, **C**)

Review of MR imaging identified secondary causes for reduced olfaction in one case (10%), there was evidence of abnormal nasal mucosal appearance on MRI in two cases but was minimal/mild and thus considered insignificant to contribute to olfactory dysfunction. There was evidence of microvascular disease in three cases. There were no significant findings on review of cribriform plate; evidence of prominent mesial temporal lobe without diffusion and subtle and non-specific mild swelling and signal abnormality in the left amygdala and hippocampus were identified in one case. No structural secondary causes for hearing loss were evident. The anatomical distribution of haemosiderin is shown in Table 1. Eight out of ten (80%) participants had involvement of at least one olfactory area (Table 1, Fig. 2) [15, 16].

## Discussion

In a cohort of patient with iSS, using a well validated and standardised smell test (UPSIT) we found that olfactory function was reduced in all participants and was significantly worse than matched normative values, and consistent with moderate microsmia or complete anosmia in 60%. This indicates a high prevalence of olfactory hypofunction in iSS. Our findings suggest that impairment of olfactory function may be a core feature of the clinical syndrome of iSS which may be relevant for the diagnosis and management of this rare but potentially disabling disease. We hypothesise that olfactory loss may be under-reported in iSS. It is not uncommon for patients to be unaware of their olfactory deficits, and in our cohort only 40% of participants were symptomatic which is similar to the findings from a study of patients with traumatic olfactory nerve injury of whom 60% were unaware of their olfactory deficits [17].

Lower (worse) UPSIT scores strongly correlated with higher (worse) hearing thresholds, even when adjusted for risk factors for hearing loss. This is likely due to similar and possibly synchronous pathophysiological processes affecting both CNVIII and the olfactory tract and bulb of CNI [6, 7, 11]. Involvement of these structures is likely at the level of the cribriform plate (CNI) and at the cerebellopontine angle (CNVIII) where they are exposed to haemorrhagic CSF along nerve sections that have an abundance of glia able to break down haem and synthesise ferritin required for iSS pathogenesis [2, 6, 7, 11, 12, 18]. Involvement of the cranial nerve central components within the brain parenchyma is unlikely—as exposure to blood products within the circulating CSF is necessary for siderosis development [6, 7, 11]. Gliosis and neuronal damage can occur in iSS as a result of lipid peroxidation from neurotoxic iron species which may inadvertently affect the olfactory axons. Involvement of Sylvian fissures may also contribute to reduced olfactory and auditory functions [2, 6, 7]. Involvement of other cranial nerves including optic nerve, with associated functional deficits, has also been reported albeit less frequently [2, 3, 19, 20].

Cognitive and olfactory function have been previously shown to be related possibly due to involvement of shared neuronal pathways in several neurodegenerative conditions including Parkinson’s disease and multiple sclerosis; studies also demonstrated that olfactory dysfunction may predict cognitive impairment in some of these disorders [21–24]. Cognitive dysfunction was previously reported in 50% of individuals with iSS and therefore was included in our analysis [1]. Overall, cognitive impairment was present in all seven participants tested (100%), with a similar pattern of affected domains to that previously reported [1]. In contrast to other studies, we did not observe an association between UPSIT scores and the number of impaired cognitive domains in our cohort, suggesting potentially that these two symptoms can occur independently.

We found that in 80% of participants haemosiderin was observed in at least one area involved in olfaction, although the anatomical extent of siderosis has not yet been validated as a marker of clinical functional status in iSS [16, 22].

The mean UPSIT scores observed in our cohort were comparable with the scores observed in individuals with mild cognitive impairment, multiple system atrophy, spinocerebellar ataxia (type 7), frontotemporal dementia and corticobasal degeneration [25].

The strength of this study is that—to the best of our knowledge—it is the first study to systematically and quantitatively assess olfactory dysfunction, determine its prevalence in iSS, and compare it to auditory and cognitive functions whilst controlling for potential confounding risk factors, and reporting on the anatomical distribution of superficial siderosis.

There are several limitations to our study, including presence of risk factors for olfactory dysfunction (other than iSS) in several of our participants, the study’s small sample size which may have also impacted the statistical analysis, and thus the results should be interpreted with caution. Other limitations include lack of study control group, unsupervised test administration, availability of cognitive assessments for some but not all participants and absence of dedicated (thin slice) MRI to visualise the olfactory bulb. The MRI protocols were not described, as several machines from different vendors are in clinical use at our Trust, or imaging had been performed externally. MR changes suggestive of sino-nasal disease were identified in one participant which may have significantly affected the participant’s olfactory function resulting in low UPSIT score.

## Conclusion

Our study demonstrates that olfactory dysfunction is common in iSS. Its prevalence and correlations between UPSIT scores and pure-tone averages suggest that olfaction, like hearing, may be a core feature of iSS, although our study results should be interpreted with caution in view of several limitations and need to be corroborated or revoked with further and larger prospective studies.

## Data Availability

Fully anonymised study data not published within this article will be stored in the affiliated data repository (https://doi.org/10.5522/04/201518120) in line with the funder’s and institutional research data policy and GDPR (2018). The principal author takes full responsibility for the data, the analyses and interpretation, and the conduct of the research.
